# Overview of the protein-protein interaction annotation extraction task of BioCreative II

**DOI:** 10.1186/gb-2008-9-s2-s4

**Published:** 2008-09-01

**Authors:** Martin Krallinger, Florian Leitner, Carlos Rodriguez-Penagos, Alfonso Valencia

**Affiliations:** 1 Structural Biology and BioComputing Programme, Spanish Nacional Cancer Research Centre (CNIO), C/Melchor F. Almagro, 3, E-28029 Madrid, Spain

## Abstract

**Background:**

The biomedical literature is the primary information source for manual protein-protein interaction annotations. Text-mining systems have been implemented to extract binary protein interactions from articles, but a comprehensive comparison between the different techniques as well as with manual curation was missing.

**Results:**

We designed a community challenge, the BioCreative II protein-protein interaction (PPI) task, based on the main steps of a manual protein interaction annotation workflow. It was structured into four distinct subtasks related to: (a) detection of protein interaction-relevant articles; (b) extraction and normalization of protein interaction pairs; (c) retrieval of the interaction detection methods used; and (d) retrieval of actual text passages that provide evidence for protein interactions. A total of 26 teams submitted runs for at least one of the proposed subtasks. In the interaction article detection subtask, the top scoring team reached an F-score of 0.78. In the interaction pair extraction and mapping to SwissProt, a precision of 0.37 (with recall of 0.33) was obtained. For associating articles with an experimental interaction detection method, an F-score of 0.65 was achieved. As for the retrieval of the PPI passages best summarizing a given protein interaction in full-text articles, 19% of the submissions returned by one of the runs corresponded to curator-selected sentences. Curators extracted only the passages that best summarized a given interaction, implying that many of the automatically extracted ones could contain interaction information but did not correspond to the most informative sentences.

**Conclusion:**

The BioCreative II PPI task is the first attempt to compare the performance of text-mining tools specific for each of the basic steps of the PPI extraction pipeline. The challenges identified range from problems in full-text format conversion of articles to difficulties in detecting interactor protein pairs and then linking them to their database records. Some limitations were also encountered when using a single (and possibly incomplete) reference database for protein normalization or when limiting search for interactor proteins to co-occurrence within a single sentence, when a mention might span neighboring sentences. Finally, distinguishing between novel, experimentally verified interactions (annotation relevant) and previously known interactions adds additional complexity to these tasks.

## Background

Physical protein-protein interactions have been studied extensively because of their crucial role in controlling central biological processes such as cell division and their implications in a range of human diseases including cancer. A collection of experimental techniques is available to characterize protein-protein interactions; some of them are more suitable to determine stable complexes whereas others are generally considered better for detecting transient interactions. The use of large-scale proteomics approaches for experimentally obtaining protein interaction information has resulted in an additional source of interaction data. Also, bioinformatics techniques based on sequence, structural, or evolutionary information have been devised to predict binary protein interactions.

To capture and provide efficient access to the underlying information, structured interaction annotations have been stored in public databases. These databases vary in annotation depth and type of interactions, but a common characteristic is that the annotations are primarily extracted by human curators from relevant publications. Some interaction databases such as the human protein-protein interaction database HPRD (Human Protein Reference Database) [1], HomoMINT (inferred human network) [2], and MIPS (Munich Information Center for Protein Sequences) [3] focus on certain taxa and store mainly information for human or mammalian proteins. There are also more specialized interaction databases like PDZBase [4], which is restricted to proteins with a PDZ domain, or Reactome [5], which focuses on interactions related to biological pathways. The interaction databases MINT (Molecular Interactions Database) [6] and IntAct [7] contain the largest number of nonredundant direct human protein-protein interactions, exceeded in number only by HPRD. They also provide literature references relevant to the individual interactions, together with the experimental interaction detection method used as supporting evidence [8].

Although most interaction databases provide links to SwissProt identifiers for their interactor proteins, to compare annotations derived from different databases as well as to share the annotation effort, both a standard annotation format as well as controlled vocabulary terms describing the experimental context are crucial. The Proteomics Standards Initiative Molecular Interaction (PSI-MI) standard has been developed to facilitate a coordinated annotation effort for protein interactions using controlled vocabulary terms and providing a common format as framework [9]. More efficient retrieval systems of biological interactions contained in scientific articles are in demand not only for specialized users such as biological database curators, but also for the general biology community. In order to improve the efficiency of locating curation relevant articles by the Biomolecular Interaction Network Database (BIND) curators, an extraction system called PreBIND based on support vector machine (SVM) classifiers to detect interaction-relevant articles, has been developed [10].

A range of methods have been proposed to extract biological associations from the literature. Some of them obtain general associations, whereas others focus on certain biologically relevant association categories (for example, protein interactions, genetic interactions [gene regulation], or gene product-functional keyword association [functional annotations]) [11,12].

In general, two baseline approaches to extract biological relations may be identified, although many of the previously published systems are actually hybrid strategies combining features from both. The approaches are termed local association analysis and global association analysis.

The local association analysis or article-centric approach tries to extract binary interactions for proteins co-occurring in a predefined textual context, often corresponding to sentences or text passages. To determine whether the co-occurring entities exhibit an interaction relationship, additional contextual characteristics are considered. Some of these rely, for instance, on the use of interaction keywords, verbs or semantic frames, or on machine learning techniques for classifying sentences according to their interaction relevance or even exploitation of syntactical rules for detecting interaction relations. Some of these approaches integrate modules that handle negation ('A [does not] interact with B') that reverse the meaning of a predicate. Other approaches can detect enumerations of multiple protein mentions in a single sentence. The advantage of the article-centric approach is that it often provides interaction-relevant sentences useful for human interpretation and can support detection of novel protein interactions with only single citation evidence. Because this type of approach extracts direct interactions together with the supporting textual evidence, it may serve to improve annotation consistency as well as facilitate annotation update.

Global association analysis, or multi-document interaction extraction, tries to exploit recurrent co-occurrence of proteins within a collection of documents or passages in order to detect protein interaction pairs [13]. The strength or reliability of the extracted interaction pairs can be calculated based on statistical co-occurrence analysis. An interaction network can thus be extracted providing a global systems biology overview that also captures indirect relations that go beyond a single document. This strategy is more suitable for capturing commonly known protein interactions, which have been extensively studied with a collection of supporting citations. One disadvantage of this approach is that it is not straightforward for human interpretation.

Most of the implemented methods extract interaction information from PubMed abstracts and titles only, and not from the corresponding full-text articles, obtaining results that are not directly comparable with the information contained in interaction databases, which access the whole documents. Another limitation is that most of them address only very narrow aspects of the interaction annotation pipeline, which gives somewhat artificial results that do not scale up to handle real applications. Among the aspects often neglected are the intricacies of the initial article selection process, as well as the linking of the interactor proteins to their corresponding database identifier, a step that is often referred to as 'normalization'. Moreover, no previously reported text-mining strategy distinguishes between experimentally verified interactions and interaction statements that lack experimental confirmation. This aspect is crucial, because most of the biological annotation databases provide an evidence qualifier for each annotation record. For BioCreative II, we developed a text-mining task for the extraction of protein-protein interaction annotations from the literature, and evaluated the submissions against a manually curated 'gold standard' carried out by expert database annotators.

The main aims posed when devising the protein-protein interaction (PPI) task were as follows.

1. Determine the performance of state-of-the-art text-mining tools in extracting PPIs, as compared with manual curation.

2. Provide participating systems with useful resources for training and testing protein interaction extraction systems.

3. Explore which approaches are successful and practical.

4. Analyze the main difficulties and aspects influencing performance of PPI extraction systems.

5. Promote the development of useful tools to extract protein-protein interactions from text.

Previously published protein interaction extraction systems do not have directly comparable evaluation setups to allow one to carry out consistent comparison and benchmark studies, and they also yield results that are not comparable with results of manual database annotation efforts (mainly due to the lack of interactor protein normalization). The BioCreative II PPI task was carried out to allow comparison of various strategies on a common benchmark dataset based on data collections prepared by domain experts and in line with the content and annotation strategy of interaction databases.

### Protein-protein interaction task

The PPI task comprised four sub-tasks, each of which was concerned with a particular aspect of the interaction annotation pipeline.

1. Interaction article subtask (IAS): classification and ranking of PubMed abstracts, based on whether they are relevant to protein interaction annotation or not.

2. Interaction pair subtask (IPS): extraction of binary protein-protein interaction pairs from full-text articles. Proteins are annotated with their corresponding unique SwissProt identifier.

3. Interaction method subtask (IMS): extraction of the interaction detection method used to characterize the protein interactions described in full-text articles. The interaction detection methods must be characterized in terms of corresponding MI ontology identifiers. They constitute the experimental evidence for the interaction.

4. Interaction sentences subtask (ISS): retrieval of the textual evidence passages that describe/summarize the interaction.

**Figure 1:**
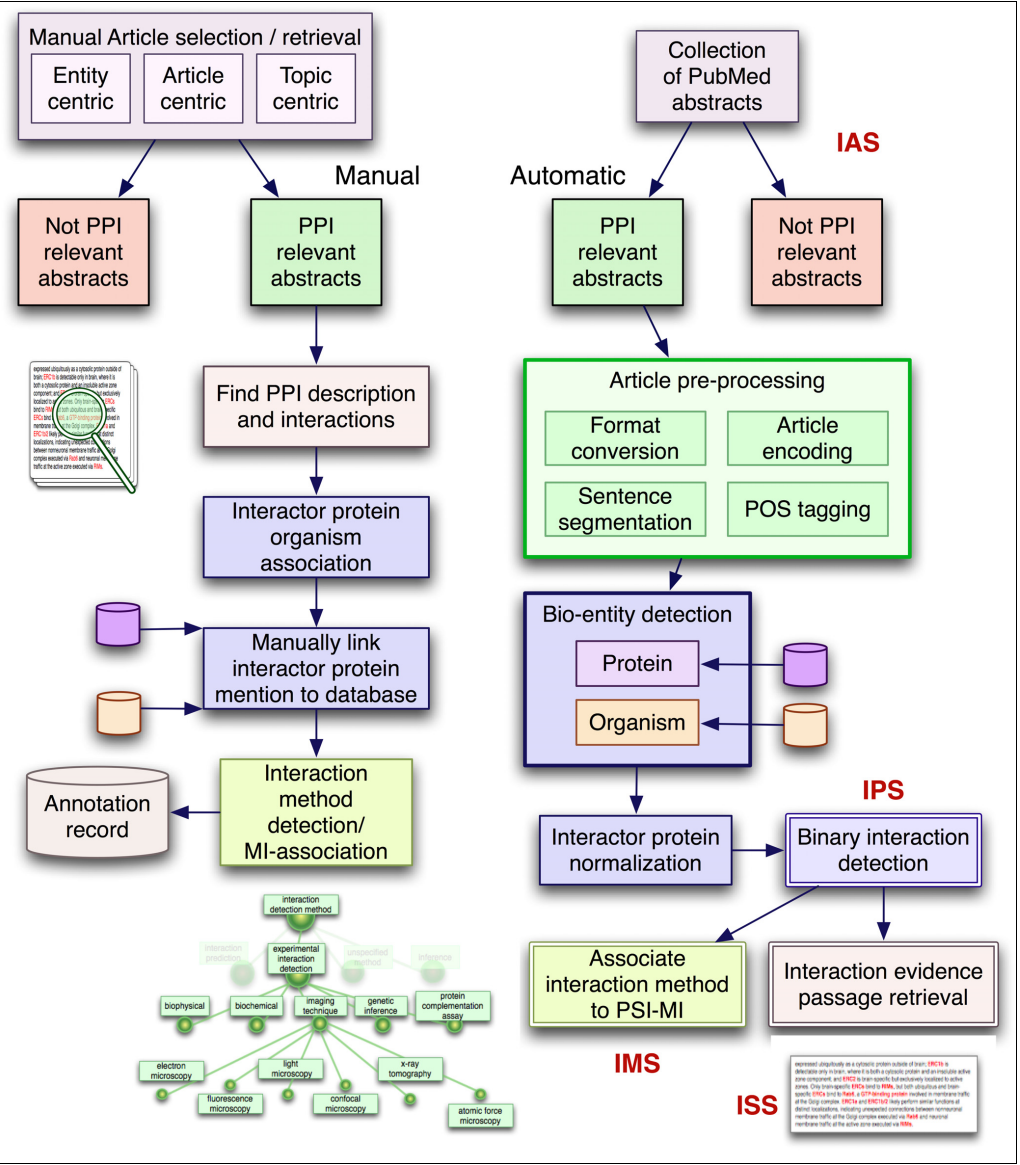
Manual versus automated protein-protein interaction annotation. Presented is a comparison between the manual protein-protein interaction (PPI) annotation process and the automatic extraction of protein interactions in the context of the PPI task of BioCreative II.

Participating teams were provided with a collection of training data for each subtask to build and train their literature mining systems during the period from June to October 2006. Then, during the test phase, the participants had to provide submissions for at least one of the PPI subtasks within a predefined, short period of time (<2 weeks, to minimize the possibility of a manual annotation attempt). Figure 1 provides a comparative flowchart of the manual protein-protein interaction annotation process with the automatic text-based extraction within the context of the BioCreative II PPI task.

## Results

A total of 26 teams submitted results for one or more PPI subtasks. Each team could provide up to three runs (submissions) per subtask, to allow them to explore different parameters or methods. The most relevant results are described in this article; additional results and data analysis are available online [14].

### Interaction articles subtask

The aim of the IAS was to determine whether text-mining tools can detect and rank interaction annotation-relevant articles based on PubMed titles and abstracts only. Although manual interaction annotations are based on inspection of full-text articles, PubMed titles and abstracts were used for this set up. This is in line with the textual data availability, because there are still general limitations in retrieving, processing, and distributing large collections of full-text articles for a considerable number of annotation-relevant biological journals. In this way we also explored the implicit limitations of abstract-based detection of annotation-relevant articles. Resulting applications would enable a more efficient retrieval of protein-protein interaction literature for biologists, as well as assisting database curators in the initial article selection step.

Most of the manual curation strategies start with initial reading of abstracts, followed by detailed examination of the corresponding full-text articles only when, based on the abstract, the article appears to be worthwhile for manual curation. The actual protein interaction annotations in turn are extracted (in most cases) from the full-text articles. This implies that there are cases where the abstracts alone are not informative enough to determine curation relevance for the corresponding full-text article. To increase recall, the MINT and IntAct databases carry out a shared, exhaustive curation effort for a specified list of journals of particular high content of protein interaction annotations. The idea behind this shared curation is to ensure that all the interaction annotation information contained in articles from a certain publication period of these journals has been extracted.

Participants received a training collection of abstracts resulting from the curation effort of the IntAct and MINT interaction databases to develop their systems. To evaluate the performance of these systems, participants received a test set of unlabeled abstracts, which they had to classify into interaction relevant or nonrelevant articles. For the evaluation, participants were asked to return two separate ranked lists of article identifiers as output, one for the abstracts classified as protein interaction relevant and one for the abstracts classified as nonrelevant. They were asked to generate these classifications automatically, without human re-ranking or manual inspection.

A total of 19 teams submitted 51 runs (up to three runs were allowed per team). The automatically classified articles were compared with the classification carried out by the interaction database curators. As evaluation measures, the recall, precision, balanced F score and area under the receiver operating characteristic curve (AUC) were used, as is customary for information retrieval experiments (see, for example, the TREC [Text Retrieval Conference] competitions [15], which have recently included a Genomics track):

$$\text{Precision}=\frac{\text{TP}}{\text{TP+FP}}$$

$$\text{Recall}=\frac{\text{TP}}{\text{TP+FN}}$$

$$\text{Fscore}=\frac{2\times \text{Precision}\times \text{Recall}}{\text{Precision}+\text{Recall}}$$

$$\text{Accuracy}=\frac{\text{TP}+\text{TN}}{\text{P}+\text{N}}$$

**Figure 2:**
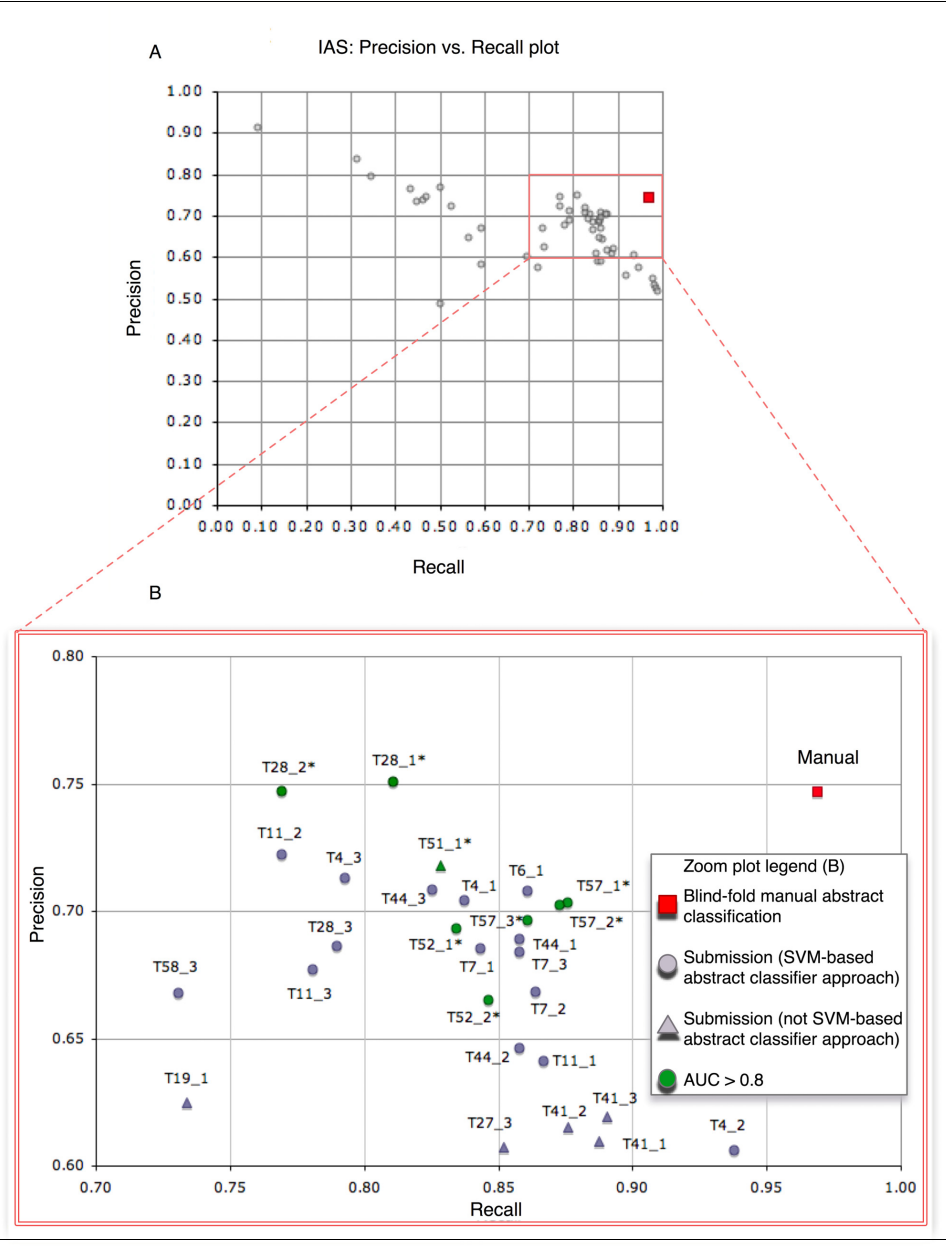
Precision versus recall plot for the IAS. **(a) **Overview plot for all of the received submissions and **(b) **zoomed view of the top scoring teams, with some additional details related to the methods used (SVM-based approaches are represented by circles, and other methods by triangles) as well as the AUC score. Runs with an AUC greater than 0.8 are shown in green. AUC, area under the receiver operating characteristic curve; IAS, interaction article subtask; SVM, support vector machine.

Where TP is number of true-positive predictions (interaction-relevant abstracts correctly identified); FP is the number of false positives (abstracts which are not interaction-relevant predicted as such by the participants); FN is the number of false negatives (interaction-relevant abstracts wrongly classified as non-relevant by the participants); TN is the number of true negatives (nonrelevant abstracts, which were identified as such by the participants); P is the total number of positives (interaction-relevant); and N is the total number of negatives (not interaction-relevant abstracts). To calculate the AUC, the standard R package ROCR, which integrates the most common evaluation metrics to assess the performance of classifiers [16], was used. Figure 2 shows the overall precision-recall plot for the IAS (part a) as well as a more detailed zoom of the top scoring teams (part b). A detailed collection of the IAS results obtained by each run of the participating teams is provided in Table 1.

**Table 1:** IAS result overview

Team	Run	Precision	Recall	F score	AUC	Accuracy
T4	1	0.7040	0.8373	0.7649	0.7495	0.7430
T4	2	0.6061	0.9379	0.7364	0.5529	0.6647
T4	3	0.7128	0.7929	0.7507	0.7479	0.7371
T6	1	0.7080	0.8609	0.7770	0.8554	0.7533
T7	1	0.6851	0.8432	0.7560	0.8270	0.7282
T7	2	0.6682	0.8639	0.7535	0.7875	0.7179
T7	3	0.6840	0.8580	0.7612	0.8318	0.7312
T11	1	0.6411	0.8669	0.7371	0.7995	0.6913
T11	2	0.7222	0.7692	0.7450	0.7567	0.7371
T11	3	0.6769	0.7811	0.7253	0.7013	0.7046
T14	1	0.7343	0.4497	0.5578	0.7500	0.6440
T14	2	0.7371	0.4645	0.5699	0.7561	0.6499
T14	3	0.7465	0.4704	0.5771	0.7570	0.6558
T19	1	0.6247	0.7337	0.6748	0.6765	0.6470
T19	2	0.6453	0.5651	0.6025	0.6765	0.6278
T27	1	0.5886	0.8550	0.6972	0.6812	0.6292
T27	2	0.5554	0.9201	0.6927	0.6244	0.5923
T27	3	0.6076	0.8521	0.7094	0.6945	0.6514
T28	1	0.7507	0.8107	0.7795	0.8471	0.7710
T28	2	0.7471	0.7692	0.7580	0.8150	0.7548
T28	3	0.6864	0.7899	0.7345	0.7993	0.7149
T30	1	0.5826	0.5947	0.5886	0.6197	0.5849
T30	2	0.4871	0.5030	0.4949	0.5643	0.4874
T30	3	0.5995	0.6953	0.6438	0.6581	0.6160
T31	1	0.6678	0.5947	0.6291	0.6714	0.6499
T31	2	0.7206	0.5266	0.6085	0.6793	0.6617
T31	3	0.7959	0.3462	0.4825	0.6793	0.6292
T37	1	0.5480	0.9793	0.7028	0.6976	0.5864
T37	2	0.5755	0.9467	0.7159	0.7468	0.6248
T37	3	0.5312	0.9822	0.6895	0.6550	0.5583
T41	1	0.6098	0.8876	0.7229	0.7535	0.6603
T41	2	0.6154	0.8757	0.7228	0.7720	0.6647
T41	3	0.6193	0.8905	0.7306	0.7714	0.6721
T44	1	0.6888	0.8580	0.7642	0.7320	0.7356
T44	2	0.6459	0.8580	0.7370	0.5970	0.6942
T44	3	0.7081	0.8254	0.7623	0.7433	0.7430
T48	1	0.9118	0.0917	0.1667	0.6572	0.5421
T48	2	0.5887	0.8639	0.7002	0.6422	0.6307
T48	3	0.8346	0.3136	0.4559	0.6904	0.6263
T49	1	0.5261	0.9852	0.6859	0.7968	0.5495
T49	2	0.5170	0.9911	0.6795	0.7990	0.5332
T49	3	0.5741	0.7219	0.6396	0.5894	0.5938
T51	1	0.7179	0.8284	0.7692	0.8412	0.7518
T52	1	0.6929	0.8343	0.7570	0.8057	0.7326
T52	2	0.6651	0.8462	0.7448	0.8146	0.7105
T57	1	0.7031	0.8757	0.7800	0.8194	0.7533
T57	2	0.7024	0.8728	0.7784	0.8151	0.7518
T57	3	0.6962	0.8609	0.7698	0.8054	0.7430
T58	1	0.7656	0.4349	0.5547	0.7326	0.6514
T58	2	0.7692	0.5030	0.6082	0.7578	0.6765
T58	3	0.6676	0.7308	0.6977	0.7554	0.6839

AUC, area under the receiver operating characteristic curve; IAS, interaction article subtask.

Team 6 (Alex and coworkers [17]) achieved the highest AUC (0.8554), with a precision of 0.7080, a recall of 0.8609, and an F score of 0.7770. They applied a SVM classifier together with careful pre-processing, stemming, part-of-speech (POS) tagging, sentence splitting and shallow parsing. Team 6 also integrated a protein name detection and abbreviation resolution systems. Team 57 [18] obtained the highest F score (0.7800), also using a SVM-based text classifier. Figure 2b shows that a common characteristic among a considerable fraction of the top scoring teams was the usage of SVM techniques for their classification systems. The usefulness of SVMs to detect interaction literature had previously been explored both at the level of abstracts and at the sentence level [19]. A common trend in the submitted runs was the consistently higher recall when compared with precision. Manual blind classification of a randomly chosen subset of 412 test set abstracts by a domain expert (but without special training for interaction annotation) showed a similar outcome, with a very high recall of 0.97 and a considerable lower precision of 0.75. A detailed examination of the IAS test set predictions and the manual abstract classification showed that there are two basic types of false-positive categorizations when compared with annotations by interaction database curators. The first type refers to protein interaction-related abstracts that nonetheless do not have corresponding protein-protein interactions that are worth annotating in the full text. This implies that there are cases in which abstracts do not provide sufficient information to determine with certainty whether an experimental protein interaction characterization is described in the corresponding full-text article. The second type of false-positive prediction corresponds to abstracts describing interaction relations, but not between proteins. These included the following interaction classes.

1. Protein-DNA interaction descriptions: these mainly refer to transcriptional complexes that comprise associations between regulatory gene sequences and transcription factors (PMID 16311517, PMID 16601684).

2. RNA interaction descriptions: these refer to associations between RNA molecules (for example, tRNA interaction with the mRNA; PMID 16724118) or between proteins and RNA molecules, such as in 'Musashi interacts specifically with the polyadenylation response element in the 3' untranslated region of the Mos mRNA' (PMID 16763568).

3. Cellular and subcellular structure interaction descriptions: these refer to interactions of cells or cellular structures or between proteins and cellular structures, such as liposomes (PMID 10409698), the nuclear envelope, or telomeres (PMID 16467853).

4. Chemical substance interaction descriptions, including binding studies of metal ions (PMID 16601688), peptides (PMID 16494877), nucleotides (PMID 16678163), oligonucleotides (PMID 16794580), or proteoglycans (16511564). The following two phrases illustrate this kind of interaction expressions: 'bovine RPE65 binds iron ion' (PMID 16319067) and 'structure of a complex between Hrp1 and an oligonucleotide' (PMID 16794580).

5. Biological process association descriptions: these refer to relations of a protein with a certain biological process or pathway, such as in 'NDRG1 interacts with SIRT1/p53 signaling' (PMID 16314423).

6. Host-pathogen interaction descriptions (for example, for fungal pathogens; PMID 16263704).

7. Interactions between proteins and cells: 'The carboxyl-terminal domains 19-20 of FH interact with the major opsonin C3b, glycosaminoglycans, and endothelial cells.' (PMID 16601698).

8. Immune system interaction descriptions: these refer, for instance, to binding characterizations of allergens and immunoglobulins which are not curated by interaction databases (for example, IgE-binding of hevein; PMID 16638575).

9. Word sense ambiguity. When using words as features for a document classifier, an incorrect identification of the word sense can hamper the classification of abstracts. For instance, the word 'complex', often used to describe a structural assembly of proteins, can also denote 'having many relations' (PMID 16482221).

10. Protein names: false positives resulting from protein names containing interaction terms, for example, ATR-interacting protein (ATRIP; PMID 1667595).

In case of false-negative test set classifications, some of them corresponded to abstracts related to gene regulation but also mentioned characterizations of transcriptional protein-protein complexes. Because many of the negative training samples corresponded to gene regulation characterizations, these abstracts were harder to classify correctly. Other false-negative abstracts corresponded to very specific transient interaction types (PMID 16648821). From abstracts alone it often remains unclear whether the interaction mentioned is also experimentally characterized in the full-text article. Well known interactions that might be mentioned in abstracts are usually not experimentally characterized in the corresponding full-text article. Some of the abstracts do not explicitly state protein interactions but provide enough contextual information to make the article worthwhile for a full-text curation check.

To be of practical significance, evaluation metrics should take into consideration end user needs and the available amount of literature data. The BioCreative scenarios have from the start emphasized the actual usefulness of the results of the systems for their intended users, biologists and curators, so as to not create implausible or artificial test scenarios. In exhaustive curation, as was the case of the BioCreative test collection, a high recall is actually more desirable, in order not to miss any curation-relevant article. When considering other user scenarios, such as thematic curation against the whole PubMed database, high precision and efficient relevance ranking might have a greater practical impact.

**Figure 3:**
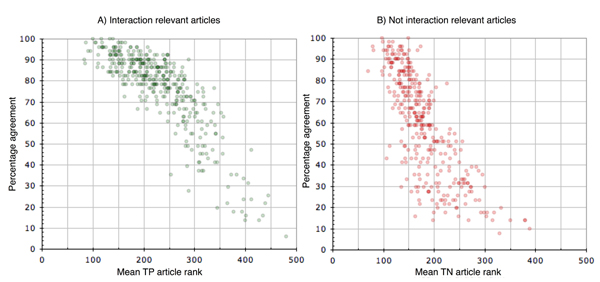
Submission agreement versus average article rank. The relation of submission agreement among different runs to the average rank of the articles is presented for both relevant and nonrelevant articles. The overall agreement between systems was lower for nonrelevant articles (*R*^2^_relevant _= 0.7 versus *R*^2^_nonrelevant _= 0.59). In general, the higher the average rank of the article, the more systems agreed on the correct classification.

To determine the effect of combining the predictions provided by different systems, a majority voting analysis was performed. Based on simple majority voting, a precision of 0.7078 can be reached. Simple majority voting based results showed a corresponding recall of 0.8817, F measure of 0.7852, and accuracy of 0.7592. The relationship between the average prediction agreement and the corresponding article rank was also studied. Figure 3 illustrates this relation, showing that the higher the agreement between different submissions on the correct class label was, the higher the corresponding average rank of this abstract. This fact supports the idea of creating an online meta-server to leverage fully the collective performance of the participating systems, as well as to enhance comparison of their individual strengths and weaknesses in real-world settings and conditions. The BioCreative meta-server is further described in another article in this supplement to *Genome Biology *[20].

### Interaction pair subtask

The IPS goes beyond previously published strategies on automatic protein interaction extraction from text. These were often characterized by strong prior assumptions, resulting in tools that were not directly comparable with existing protein interaction annotation databases and were of only limited practical use. Among the restrictions of previous efforts, as mentioned previously, is the use of abstracts only, as well as the assumption of co-occurrence of both entity mentions within single sentences. Key aspects of biological annotations - such as linking the entities to their corresponding unique database identifiers or the consideration of experimental evidence information supporting the interactions - still need further refinement to avoid piling up small individual errors into larger, more significant, global ones. Tackling these and other challenges required that participating systems use full-text articles provided in commonly available formats (PDF and HTML) and extract binary protein interaction pairs regardless of whether they co-occurred within a sentence or not. Additionally, each of the interactor proteins had to be linked to its corresponding UniProt identifier or accession number.

This implied, for instance, that for the interactor protein CARD10 the participants had to establish whether it corresponded to the human (CAR10_HUMAN) or mouse protein (CAR10_MOUSE) in order to assign the correct corresponding database identifier. The manual association of interactor proteins to their database identifiers constitutes one of the most time-consuming steps within the manual interaction curation pipeline. To develop their systems, the participants received a collection of full-text articles and the corresponding interaction annotation records of MINT and IntAct. As test collection, a set of full-text articles was released. From these, the participants had to extract the protein-protein interaction pairs for which an experimental characterization was provided in the article (for more details, refer to the data preparation section). Each of the submissions was scored in terms of precision and recall of the interaction pairs provided by the system, as compared with the manually extracted ones.

To avoid inconsistencies resulting from the use of different database releases, we provided a baseline SwissProt collection and only protein identifiers contained in that database version were considered for evaluation purposes. Nevertheless, in practice it is not always possible to normalize the interactor proteins mentioned in the text to a single database, because there is currently no biological database that covers all of the proteins described in the literature. Therefore, we analyzed separately the following groups of articles: those mentioning only interaction pairs in which both proteins could be linked to a SwissProt record (SwissProt-only article set); and those also including interactions formed by a protein contained in SwissProt and a protein contained in the TrEMBL (Translated EMBL) database only (whole article set).

When calculating the performance of the interaction extraction systems, two general evaluation strategies can be distinguished.

1. The micro-averaged performance is based on combining results from each interactor protein pair, weighting equally all pairs, regardless of the number of interactions mentioned per article. This number can vary considerably, depending on whether a high-throughput experiment or low-throughput detailed interaction characterization was carried out. This score reflects the global performance of a system when compared with the whole collection of curated interaction pairs.

2. Macro-averaged scores are based on computing results per document and then averaging them for the whole document collection. Thus, the score for each document is weighted equally.

The second evaluation strategy is more useful for practical applications, because it provides some insight into how stable the method is when it is applied to a given article. Tables 2 and 3 show the evaluation of the protein-protein interaction pair extraction for the whole test set article collection and the SwissProt-only article subset, respectively.

**Table 2:** IPS result overview (whole article collection)

Team	Run	Precision	Recall	F score
4	1	0.3893	0.3073	0.2885
6	1	0.2758	0.3011	0.2532
6	2	0.2218	0.2592	0.2066
6	3	0.2392	0.3035	0.2272
11	1	0.0510	0.2753	0.0717
11	2	0.0510	0.2753	0.0717
11	3	0.0517	0.2776	0.0726
14	1	0.1791	0.1421	0.1384
14	2	0.1944	0.1300	0.1414
14	3	0.1162	0.1057	0.0985
17	1	0.0413	0.2543	0.0631
17	2	0.1018	0.2012	0.1182
17	3	0.1633	0.2066	0.1599
19	1	0.0854	0.2115	0.1036
19	2	0.1144	0.2681	0.1361
19	3	0.1595	0.2466	0.1690
28	1	0.1373	0.2905	0.1579
28	2	0.2177	0.2651	0.2039
28	3	0.3096	0.2935	0.2623
30	1	0.0551	0.1888	0.0731
30	2	0.0345	0.2352	0.0528
30	3	0.1574	0.1846	0.1382
36	1	0.0441	0.1121	0.0503
36	2	0.0229	0.0990	0.0305
36	3	0.0548	0.1350	0.0680
40	1	0.0762	0.2489	0.0990
40	2	0.2632	0.2484	0.2171
42	1	0.0160	0.4167	0.0280
42	2	0.2384	0.2218	0.2014
42	3	0.2101	0.2024	0.1827
43	1	0.0395	0.0846	0.0424
43	2	0.0828	0.0680	0.0653
43	3	0.0620	0.0867	0.0592
47	1	0.0830	0.1891	0.0910
47	2	0.0889	0.1909	0.0950
47	3	0.0747	0.1855	0.0844
49	1	0.0109	0.1092	0.0185
49	2	0.0289	0.0557	0.0345
49	3	0.0255	0.0865	0.0357
58	1	0.0003	0.0006	0.0004
58	2	0.0003	0.0006	0.0004
58	3	0.0004	0.0006	0.0005
60	1	0.0323	0.0942	0.0362
60	2	0.0162	0.0558	0.0205
60	3	0.0251	0.0654	0.0299

Interaction pair subtask (IPS) results for the whole article set. The average precision, recall, and F score obtained for each of the test set submissions are shown.

**Table 3:** IPS result overview (SwissProt only article collection)

Team	Run	Precision	Recall	F score
4	1	0.3908	0.2970	0.2849
6	1	0.3150	0.3356	0.2871
6	2	0.2519	0.2868	0.2308
6	3	0.2632	0.3394	0.2532
11	1	0.0562	0.2850	0.0770
11	2	0.0562	0.2850	0.0770
11	3	0.0569	0.2879	0.0780
14	1	0.1975	0.1543	0.1510
14	2	0.2113	0.1430	0.1552
14	3	0.1287	0.1157	0.1079
17	1	0.0452	0.2765	0.0684
17	2	0.1138	0.2274	0.1334
17	3	0.1901	0.2396	0.1862
19	1	0.0882	0.2287	0.1092
19	2	0.1200	0.2912	0.1453
19	3	0.1750	0.2748	0.1865
28	1	0.1566	0.3189	0.1784
28	2	0.2434	0.2828	0.2247
28	3	0.3696	0.3268	0.3042
30	1	0.0624	0.2153	0.0824
30	2	0.0367	0.2533	0.0557
30	3	0.1646	0.1964	0.1468
36	1	0.0456	0.1243	0.0560
36	2	0.0202	0.0997	0.0295
36	3	0.0560	0.1362	0.0686
40	1	0.0824	0.2672	0.1083
40	2	0.2751	0.2737	0.2355
42	1	0.0177	0.4368	0.0307
42	2	0.2522	0.2331	0.2112
42	3	0.2278	0.2158	0.1970
43	1	0.0412	0.1032	0.0491
43	2	0.1032	0.0836	0.0803
43	3	0.0734	0.1082	0.0731
47	1	0.0876	0.1964	0.0931
47	2	0.0940	0.1988	0.0978
47	3	0.0791	0.1920	0.0860
49	1	0.0107	0.1085	0.0186
49	2	0.0246	0.0564	0.0319
49	3	0.0234	0.0871	0.0340
58	1	0.0000	0.0000	0.0000
58	2	0.0000	0.0000	0.0000
58	3	0.0000	0.0000	0.0000
60	1	0.0384	0.1113	0.0422
60	2	0.0179	0.0631	0.0213
60	3	0.0281	0.0686	0.0314

Interaction pair subtask (IPS) results, SwissProt only. Average precision, recall, and F score obtained for the SwissProt-only test set articles for each of the evaluated runs of the IPS.

For this subtask, a total of 45 runs from 16 teams were evaluated. As a general trend, the performance of the systems on the SwissProt-only article set was slightly higher, both in terms of recall (in case of 40 runs) as well as precision (in case of 38 runs). Looking at the performance of individual systems, the run submitted by team 4 [21] obtained the highest average precision of 0.39, followed by team 28 [22] with 0.31 and team 6 [17] reaching a 0.28.

With respect to recall, team 42 [23] submitted the top-scoring run (0.42), followed by team 4 (0.37) and team 6 (0.30). To provide a balanced view of both precision and recall, the average of the F scores obtained for the test set article was also calculated. Here, team 4 obtained an average F score of 0.29, followed by run 3 of team 28 (0.26) and run 1 of team 6 (0.25). As can be seen in Table 3 in case of the SwissProt-only article set, team 28 obtained the best average F score (0.30), followed by team 6 (0.29) and team 4 (0.28).

A common characteristic of the top scoring teams was the use of rather sophisticated interactor protein normalization strategies when compared with other systems; some of them are described in this supplement. This emphasizes the interconnectedness of the individual components of the pipeline, where correct identification of protein mentions and linkage to corresponding database records is one of the crucial aspects for subsequent successful interaction extraction. For this reason, we also analyzed the interactor protein normalization performance by comparing the list of interactor proteins extracted automatically with the list derived from manual curation.

The macro-averaged precision, recall, and F score for articles with at least a single prediction were calculated. The highest precision for correct interactor protein normalization was of 0.56 (team 4) in case of the whole article set and of 0.57 (team 28) for the SwissProt-only collection. Obviously, the recall obtained for the interactor normalization was generally higher than its corresponding precision. In case of the whole test set, team 42 had the highest recall of 0.68, followed by 0.55 of team 30 (Nakov and Divoli [24]) and 0.54 of team 11 (Abi-Haidar and coworkers [25]). When looking at the SwissProt-only set, team 42 could obtain a recall of 0.69, but with a modest precision (0.08). Considering the F scores obtained for the test set articles, team 28 reached a score of 0.52 for the SwissProt-only set and team 4 of 0.48 in case of the whole test set collection.

### Interaction method subtask

The reliability of protein interactions is strongly linked to the underlying experimental evidence providing support for a specific interaction pair. Each experimental interaction detection technique offers a certain implicit degree of reliability, sometimes also providing contextual information as to whether it is an *in vivo *or *in vitro *interaction or the corresponding basic interaction type (stable or transient). For most expert-derived biological annotations, the experimental evidence is one of the central curation criteria. To provide a consistent controlled vocabulary for describing protein interaction experiments, the Molecular Interaction (MI) ontology has been developed [26], which is part of the curation standard of both the IntAct and MINT databases. For the IMS task, the precision for each of the six runs submitted by the two participating teams was calculated. This was done by comparing the automatically extracted list of experimental techniques used to confirm protein-protein interactions described in each test set article with a previous manual annotation performed by database curators. Interaction detection methods had to be provided in the form of their corresponding unique MI concept identifier, which allows direct mapping into the MI ontology. Each concept in the MI ontology is characterized by an associated term as well as additional information such as short definitions, and a reference. A total of 874 article-molecular interaction detection method associations were provided in the test set. Taking into consideration the relations of the interaction detection concepts in the MI ontology, the evaluation metrics where calculated as follows:

1. Exact concept matching: implies that the submitted interaction detection method is an exact match with respect to the gold standard annotated concept.

2. Parent concept matching: implies that the submitted interaction detection method is either an exact match or a parent concept with respect to the gold standard annotation. A parent concept was defined as any higher node (a more general concept) in the MI ontology. Therefore the directed acyclic graph structure of the MI ontology was exploited, testing the path from the predicted concept to the annotated one.

Tables 4 and 5 show the results obtained by teams 14 (Ehrler and coworkers [27]) and 40 (Rinaldi and colleagues [28]) for exact match and parent match evaluation, respectively. The highest precision (0.67) was obtained by the first run of team 40, whereas the best F score (0.45) was obtained by the third run of the same group. Team 40 applied a pattern matching approach, automatically generating variants of the interaction method terms as provided in the MI ontology. Handcrafted patterns for some of the methods were included. When comparing the results between the two evaluation types, the average increase in precision and recall for the parent matching with respect to the exact matching was 0.13, and 0.11 in case of the F measure. The most significant performance difference in terms of F score (0.22) was for team 40 (run 2). It should be noted that team 14 only based their predictions on the abstracts, explaining their lower performance and highlighting the importance of full text usage.

**Table 4:** IMS result: exact matching

Team	Run	Precision	Recall	F score
14	1	0.3628	0.2172	0.2513
14	2	0.3186	0.1980	0.2249
14	3	0.3348	0.1938	0.2265
40	1	0.6679	0.3383	0.4207
40	2	0.4028	0.5548	0.4363
40	3	0.5068	0.5222	0.4836

Interaction method subtask (IMS) results: exact matching. The results correspond to the averages calculated after scoring each article in terms of precision, recall, and F score for the identification of exact matching of article to normalized Molecular Interaction (MI) identifiers.

**Table 5:** IMS result: parent matching

Team	Run	Precision	Recall	F score
14	1	0.4986	0.3078	0.3495
14	2	0.4471	0.2847	0.3170
14	3	0.4881	0.2953	0.3375
40	1	0.6794	0.3472	0.4302
40	2	0.5899	0.8548	0.6519
40	3	0.6541	0.7093	0.6375

Interaction method subtask (IMS) result parent matching. Same as Table 4 but using the parent matching evaluation.

### Interaction sentences subtask

The ISS was carried out because of increasing interest in retrieving informative text passages in full-text articles supporting biological annotations that can help in the curation, interpretation, and update of annotations. In full-text articles, multiple evidence passages might appear that provide protein-protein interaction evidence, some being more relevant than others to summarize a given interaction. For the ISS, participants had to provide, for each protein interaction pair, a ranked list of a maximum of five evidence passages describing their interaction. Each submitted evidence passage could comprise up to three consecutive sentences. The submitted passages were pooled, duplicates removed, and a unique identifier was assigned to each of them. The same was done for the set of best summarizing interaction evidence passages provided by the database curators.

The predictions were evaluated in terms of percentage of interaction-relevant sentences with respect to the total number of predicted (submitted) sentences. Also, the mean reciprocal rank of the ranked list of interaction evidence passages with respect to the manually chosen best interaction sentence was calculated. To determine whether the submitted passage corresponded to a passage in the gold standard collection, we applied an approach based on sliding the shorter over the longer one (after stripping any HTML tags) and calculating for each position the corresponding string similarity between both passages. Predicted passages were considered as correct (mapping to the manually curated ones) in case that the string similarity between them was significant. For calculating the string similarity the Python difflib library was used. Table 6 contains the results. Team 4 had the highest score, with 19% of passages that could be mapped to the manually extracted set. This team submitted few passages, but with a high fraction of correct ones. The score reported here was evaluated on the basis of the specific passages identified by the curators. However, it is also possible that alternative sentences appeared in the full-text article that described the interactions, but were not selected by the curator.

**Table 6:** ISS results: parent matching

Team	Run	Total	TP	Unique	TP (unique)	Percentage correct	Percentage correct (unique)	MRR
4	1	372	51	361	51	0.1371	0.1413	-
4	2	372	71	361	70	0.1909	0.1939	-
6	1	2,497	147	2,072	117	0.0589	0.0565	0.5525
11	1	18,385	360	5,156	131	0.0196	0.0254	0.6594
11	2	18,371	376	5,270	145	0.0205	0.0275	0.6253
11	3	18,371	387	5,252	156	0.0211	0.0297	0.6416
14	1	634	13	579	12	0.0205	0.0207	0.8718
14	2	458	10	422	10	0.0218	0.0237	0.8167
14	3	560	13	514	11	0.0232	0.0214	0.8718
27	1	1,420	37	1,386	36	0.0261	0.0260	0.4653
28	1	3,028	150	3,001	148	0.0495	0.0493	0.3740
28	2	2,249	127	2,231	126	0.0565	0.0565	0.3696
28	3	5,448	352	3,210	191	0.0646	0.0595	0.3392
36	1	4,515	232	3,407	169	0.0514	0.0496	0.5731
36	2	11,827	571	7,526	343	0.0483	0.0456	0.5813
36	3	4,083	247	3,018	161	0.0605	0.0533	0.5476
43	1	3,691	111	3,117	97	0.0301	0.0311	0.4083
43	2	1,507	69	1,383	63	0.0458	0.0456	0.3524
43	3	3,674	148	3,257	131	0.0403	0.0402	0.3449
47	1	7,934	278	4,975	159	0.0350	0.0320	0.5232
47	2	7,633	274	4,835	156	0.0359	0.0323	0.5205
47	3	8,355	290	5,172	163	0.0347	0.0315	0.5329
49	1	21,431	590	10,422	285	0.0275	0.0273	0.3785
60	1	2,243	104	2,019	91	0.0464	0.0451	0.3460
60	2	4,714	157	3,932	130	0.0333	0.0331	0.3959
60	3	7,780	229	6,293	192	0.0294	0.0305	0.3998

This table reflects the baseline evaluation of the submissions received for interaction sentences subtask (ISS). Here, the submitted passages were compared with the previously manually selected passages reflecting the best interaction evidence. The 'Total' column indicates the total number of evaluated passages (note that submissions of articles for which the curators could not find a suitable evidence passage where excluded from evaluation). True positive ('TP') is number of correct passages (mapping the manually annotated ones). The 'Unique' column shows the number of unique passages per run (after removing duplicate passages). The 'TP (unique)' column indicates the number of correct passages (mapping the manually annotated ones) in the collection of unique passages. 'Percentage correct' is the fraction of predicted passages corresponding to the 'best' previously extracted passages. The 'Percentage correct (unique)' is the fraction of unique predicted passages corresponding to the 'best' previously extracted passages. 'MRR' is the mean reciprocal rank of the correct passages. Note that in the case of team 4, the MRR should not be taken into account, because all of the submitted passages here were labeled with rank 1 by this team.

Most abstract-derived interaction sentences in the training data collections provided for this task lacked experimental information, which made the extraction of correct passages more challenging.

The passages extracted by database curators often mentioned the experimental detection method used to characterize the described interaction or a reference to figures where the experimental outcome was shown (80% of the cases), whereas passages submitted by participating teams often did not mention any interaction experiment. For example, the following is a sentence extracted by the curators that reflects this aspect: 'HAX-1 co-immunoprecipitates with BSEP, MDR1, and MDR2 from transfected cells and hepatocytes.' Here, 'co-immunoprecipitates' implies that a co-immunoprecipitation experiment was done, which confirmed the interaction between HAX-1 and BSEP, MDR1, and MDR2

The ISS top performing team 4 applied multiple techniques to retrieve interaction passages: the location of the sentence in the document, the relation with figures and tables, whether interaction-indicating keywords were present, the mention of experimental methods, as well as summary-indicating cue words.

## Discussion and conclusion

The PPI task of the second BioCreative challenge was designed to cover the main aspects relevant to automatically extracting biological annotations from the scientific literature, namely normalized and experimentally verified protein interactions. It also reflected the importance of collaborative efforts between domain experts, who manually curate biological relevant information from the literature, and the text-mining community.

The results of the IAS task are promising and show that, in general, the detection of protein-interaction relevant articles from PubMed titles and abstracts can be achieved. A comparison with systems using the corresponding full-text articles is currently missing, but would certainly show better the boundaries of abstract-based interaction article classification. Similar systems could in principle be adapted to assist biologists in certain steps within the curation process for other biological annotation types, such as gene regulation or cellular localization of proteins.

A deeper analysis of the evaluated results showed some of the inherent challenges when using abstracts alone as well as the difficulty in constructing a suitable true-negative training set that does not present a bias because of the journal selection. In the case of articles with a high percentage of true-positive predictions, the titles and abstracts were in general characterized by a high density not only of words or expressions related to protein interactions such as 'interacts', 'binding', 'interacting partner', or 'interaction of', but also mentioned the actual names of the methods used to characterize these interactions experimentally. In the case of the test set article with PMID 16828757, expressions such as 'yeast two-hybrid screen', 'co-immunoprecipitation', and '*in vitro *binding assays' were present.

Many of the false-negative articles corresponded to cases in which gene regulation or gene expression mechanisms were mentioned. These abstracts are often relevant to both protein interactions as well as for genetic interactions. For example, the article with PMID 16547462 describes oligomeric transcription factors.

As for false-positive articles, several general characteristics can be distinguished. Surprisingly, some systems recurrently mentioned certain well characterized hub proteins, such as EGF or EGFR (for instance, PMID 16316986). One potential reason for this might be that they are often mentioned in the positive training collection. The average performances of participating systems over full-text articles, as well as the limitations when using text-mining techniques to recover such protein-protein interactions, have been explored in these tasks. Although the initial results are promising, they also indicate that certain components still need further improvements. Participating groups encountered several obstacles that increased the difficulty of detecting normalized interaction pairs from full-text articles. Some of these are listed below.

1. Errors resulting from conversion of PDF or HTML formatted documents to plain text, such as page break errors, wrong special character handling, and word joining.

2. Sentence boundary detection errors and difficulties in processing tables and figure legends.

3. Multiple organism mentions and the resulting inter-species ambiguity for protein normalization.

4. Incompleteness of currently available protein normalization resources. Existing annotation databases such as SwissProt do not contain all of the symbols or names for proteins described in the literature.

5. Difficulties in extracting the associations and in the handling of coordination (multiple interaction pairs) from a single sentence.

6. Interaction evidence phrases in legends or titles that often do not correspond to grammatically correct sentences.

7. Heavy use of domain specific terminology, for instance in the case of experimental descriptions.

8. Evidence for interactions contained in sentences that are not necessarily consecutive.

9. The need to use domain expert inference and bioinformatics tools to perform protein normalization in order to normalize some of the interactors.

10. Errors in shallow parsing and POS-tagging tools trained on general English text collections, when applied to the specific expressions and abbreviations found in biomedical texts.

**Figure 4:**
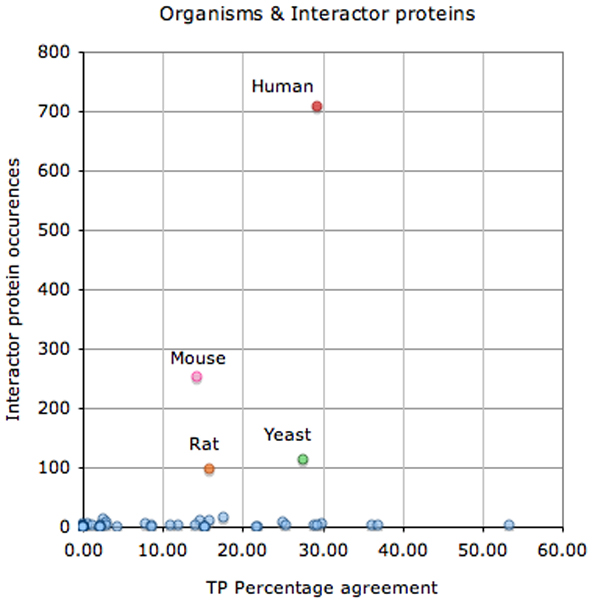
Agreement of TP interactor protein normalization versus total number of protein occurrences, by corresponding organism source. This figure shows the total number of interactor proteins in the test set for each organism with respect to the percentage agreement between different participating systems in case of the correct (true positive [TP]) predictions. Each circle represents the interactor protein set for a single species in the test set. Human (red circle), mouse (pink circle), rat (orange circle), and yeast (green circle) proteins are the most frequent interactor protein organism sources in the interaction pair subtask (IPS) test set collection. A total of 50 different organisms were included in the test set (considering the SwissProt subset), corresponding to 1,110 unique interactor proteins.

Figure 4 shows that the interactor normalization of human and yeast proteins was better than for mouse or rat proteins (it is easier to relate protein names of humans and yeast to database entries than is the case for mouse or rat).

One must keep in mind that the results of the interactor protein normalization are not directly comparable with the performance of the protein normalization tasks of BioCreative I and II, because of basic differences in the task design.

**Figure 5:**
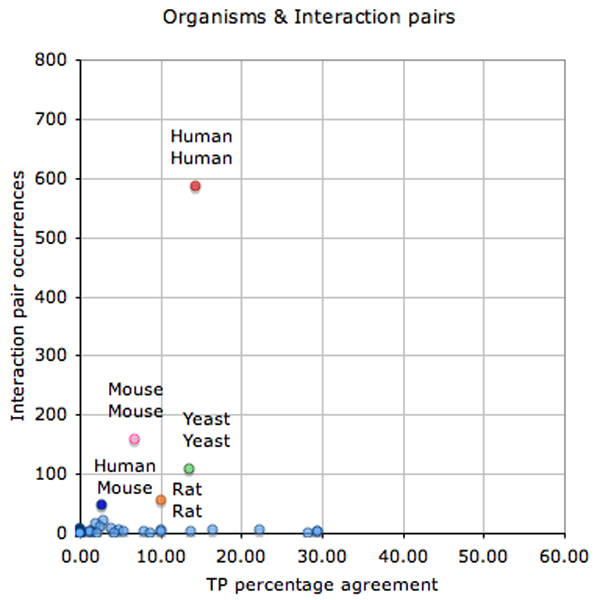
TP interaction pair normalization with respect to organism source composition. This figure shows the total number of interaction pairs in the test set (840) for each corresponding organism source combination with respect to the percentage agreement between different participating systems in case of correct (true positive [TP]) predictions. Interaction pairs of proteins form different organisms (for example, between mouse and human proteins) are basically experimental *in vitro *interactions.

This is also true for interaction pairs (Figure 5). Specifically, the correct extraction of pairs of proteins derived from different organisms is especially challenging, because it requires associating correctly each of the interactors with a different species, a process that can affect significantly both the recall as well as the precision of interaction extraction systems. Some participating teams tried to overcome the difficulty in detecting these *in vitro *interaction types by restricting their systems to interactions between proteins from the same organism source. Some participants also did not handle the extraction of homo-multimeric interaction types. For instance, the extraction of homo-dimers often requires different approaches because they are often not based on the co-occurrence of two protein mentions but rather on the presence of specific expressions such as 'dimer' or 'complex'.

The percentage of agreement of the submitted runs on the true-positive interaction pairs for interactions between proteins of different organisms was only 2.6%, as compared with 12.3% for interactions of proteins from the same species. This agreement was even lower for homo-dimeric interactions (0.85%) when compared with hetero-dimeric interactions (11.3%).

It is generally assumed that large training and test collections of full text articles with in-depth annotations of biologically relevant information will improve the performance of text-mining technologies. The data collections derived from this BioCreative PPI task can be seen as a contribution in this direction, being a useful resource for the development of interaction extraction systems. This is also true for protein mention and normalization components, where the BioCreative I challenge has already provided useful resources for abstract processing.

As a general observation on the outcome of the strategies used, it can be stated that the most sophisticated and complete systems did significantly outperform more basic strategies, which often only adapted existing supervised learning modules for this task. In case of the top performing teams, such as teams 4, 6, and 28, both general language as well as domain-specific resources were exploited. It is therefore clear that using sophisticated gene mention and normalization detection strategies generally improved the results of participating teams and constitute one of the most important components for interaction extraction systems. Also, efficient handling of linguistic coordination is crucial when extracting associations such as protein-protein interactions. The use of a supervised learning-based sentence classifier and the detection of interaction method names also seemed to play a role in the performance of interaction detection strategies.

When comparing the performance of the interactor protein normalization and the interaction pair extraction, it seems that the extraction of the interaction pairs is slightly better than would be expected. Even if details are still unclear, it might indicate a gain from the global information contained in the articles.

However, one aspect that has not been addressed in the current BioCreative evaluation is how the resulting systems would perform when doing interactive evaluation as part of curation-assistance tools. To close the gap between text-mining systems and the actual end users, such interactive assessments would be especially useful. Here, aspects such as interaction ranking, and time spent per curation when using the text mining systems compared with baseline PubMed search-based approaches, could provide additional insights into the importance of literature mining applied to the biomedical domain.

From the results obtained in the PPI task it becomes clear that although current technology might be sufficiently robust for detecting binary interactions statements from PubMed abstracts sentences, the automatic extraction and normalization of novel experimentally characterized interactions from full-text articles still requires substantial improvements in terms of performance. The strategies that participated in the PPI task can provide useful results for assisting biologists and database curators in the retrieval process of experimentally generated interaction information contained in the literature. One potential evaluation set up that could facilitate the improvement of current protein interaction annotation extraction systems would consist of aligning the different tasks that influence the interaction extraction pipeline by using a common reference data collection for all tasks and allowing specific evaluation of each individual components that influence the overall performance. Additional aspects of interest for future community evaluations in biomedical text mining are related to both qualitative as well as quantitative characterizations of the workload associated with each of the individual steps underlying manual curation, from the initial article selection to the completed annotation record, and how text-mining tools could improve the efficiency within each of these steps when compared with a baseline system of un-assisted curation. This would require available online systems that could be directly tried out by the potential end-user community.

## Materials and methods

### Data and corpus collections

#### Interaction article subtask datasets

The submissions had to be made in a predefined format, together with a short system description. The construction of a suitable training set for the IAS exploited the content of existing interaction databases, namely IntAct and MINT. The motivations behind this data selection strategy were the following:

1. Explore the usability of existing citation collections derived from biological annotation databases for the detection of curation-relevant articles.

2. Pinpoint the main challenges for selecting and retrieving suitable article collections, based on existing database citations.

3. Evaluate the use of abstract-based article classification and ranking versus manually curated articles.

The annotation records of both interaction databases are freely accessible for download and share a common annotation standard based on the HUPO (Human Proteome Organisation) PSI-MI format. The training collections were distributed using a simple XML-like format. Three abstract collections were included in the training set for this subtask.

1. The positives collection (physical protein-protein interaction relevant articles) was based on a set of PubMed articles that are relevant for protein interaction curation in the sense of the annotation process and guidelines used by the MINT and IntAct databases. This means that the corresponding full-text articles have been used to extract manual annotations and therefore meet the underlying curation standards used to extract experimentally verified protein interaction information. The initial collection contained articles resulting from exhaustive curation as well as from thematic curation. Some articles were removed from this collection mainly because either no corresponding abstracts could be retrieved from PubMed or because they corresponded to results obtained by large-scale experiments. Because the actual curation was done based on the full-text articles as opposed to the abstracts, it is conceivable that in some cases the abstracts in this collection may lack sufficient information to be considered interaction-relevant. The initial positive training collection consisted of 3,536 PubMed titles and abstracts distributed together with the corresponding PMID and the article source (journal and publication date).

2. The negatives collection (nonrelevant articles) consisted exclusively of journal titles and abstracts rejected during exhaustive curation. These articles have no associated annotation records extracted by the domain expert curators and are thus not relevant to protein interaction annotation (negative training instances were available only for those journals for which exhaustive curation had been carried out). The negative collection contained a total of 1,959 entries. The training collections of the positive and negative instances were not balanced; participating systems had to address the resulting class imbalance.

3. Finally, we also included a collection of likely positive articles, consisting of PubMed citations that had been extracted from protein interaction annotations curated by other interaction databases (including BIND, HPRD, MPACT, and GRID). This additional large collection constitutes a noisy dataset, in the sense that the corresponding databases have different annotation standards compared with MINT and IntAct (for instance, regarding the curation of genetic interactions) and thus have not been included as part of the ordinary positive training collection. This collection consisted of a total of 18,930 records.

No restrictions in terms of using additional resources or data collections for the purpose of system development and training were imposed on the participating teams. Therefore, additional resources, such as those resulting from gene mention detection or associated MeSH terms could be exploited, as is also done in real-life situations.

In order to perform a comparative assessment of the various participating systems, a common test data collection was provided to all participants. This dataset consisted of a collection of PubMed records (article titles and abstracts) in a format compliant with the training collection, but without providing the corresponding article source information as well as without the actual class label (relevant or not relevant). Most of the articles in the test set resulted from exhaustive curation of recent publications from specified journals (such as the *EMBO Journal *or *FEBS letters*) published over a predefined period of time. The resulting annotations from the curation of these articles were held back by the interaction databases until the competition was over. Some of the initial test set articles supplied by the database curators had to be removed from the test set, because no PubMed abstract was available. An additional criterion for the construction of the test set was to make sure that neither publication date nor journal name could be used as a relevant discriminative feature for classifying the articles.

The relevant and nonrelevant entries were randomly shuffled so that the article order in the test collection could also not be used to differentiate relevant from nonrelevant records. The resulting test set collection of 750 entries was an actual subset from the initial collection provided by the database curators. One of the databases also provided a small number of un-curatable abstracts, meaning that the associated full-text articles were not worthwhile to curate (too complicated and from a very specific scientific subdiscipline) or the abstract was misleading, meaning that protein interactions were mentioned in the abstract but the full-text article lacked the experimental characterization for the proposed interactions. These articles were also removed from the test collection.

The resulting initial IAS test set consisted of 375 positive (relevant) and negative (nonrelevant) entries, respectively. Nevertheless, during the postevaluation period, several records were revised and finally removed from the initial test collection. Thus, the revised test set contained a slight imbalance, consisting of 338 interaction relevant articles and 339 nonrelevant records (for a total of 677 instances).

#### Interaction pair subtask, interaction method subtask, and interaction sentences subtask datasets

For these subtasks a larger training collection of full-text articles (740) and a smaller collection of test set articles (358) were provided to registered teams. Both collections contained full-text articles in different formats, namely as HTML and PDF. Additionally, we also provided these articles as plain text automatically converted from HTML to plain text using html2text and from PDF to plain text using pdftotext. Both collections consisted of subsets of the original training and test set provided by the interaction databases after extensive filtering. For the subselection process, the following criteria were taken into account.

1. Redundancy: duplicate articles that had been annotated by both databases were removed.

2. Journal: only articles from publishers who made full-text available could be included for this evaluation.

3. Large-scale experiments: articles that mentioned large-scale experiments were removed.

4. Full text: only full articles which were currently available both in HTML and PDF formats were included; in case of articles published before 2000, the full-text articles were often only available in PDF.

5. Format: in some cases, the articles could not be converted to plain text using the previously mentioned tools and had to be removed.

For the training package, in addition to the 740 full-text articles in the various previously mentioned formats, the associated annotation files for each article in standard PSI-MI format and as flat annotation files were provided to the participants. These annotations contained the normalized interaction pairs, the interaction detection methods, as well as some additional information curated by the interaction databases. Also, a file with the MI identifiers of concepts that were children or ancestor nodes of the interaction detection method (a total of 155 concepts) formed part of the training package for the IMS.

In case of the ISS, only a limited amount of unique full-text interaction evidence passages could be provided for the training collection (63). To compensate, additional resources were included in the training package:

1. Anne-Lise Veuthey corpus: a collection of sentences kindly provided by Anne-Lise Veuthey from the Swiss Institute of Bioinformatics (SIB), containing protein interaction related sentences from PubMed abstracts. It included a total of 697 evidence sentences.

2. Prodisen interaction subset: a collection of 921 sentences related to interactions derived from the Prodisen corpus [19]. Each sentence from a given abstract was manually classified regarding whether it contained interaction descriptions of genes and proteins.

3. Christine Brun corpus: a set of sentences derived from abstracts related to interactions and their corresponding interaction type (defined as direct or indirect).

4. GeneRIF interactions: the collection of interaction sentences provided by GeneRIF. There are a total of 51,381 entries in this collection.

Although all of these additional collections are related to interaction sentences, they differ from the passages extracted by the interaction database curators in several points: they are derived from abstracts alone, whereas the BioCreative interaction evidence passages were extracted from full-text articles; and they are single sentences, whereas the BioCreative test passages can span several sentences.

The training data for the IMS consisted of a subset of annotations and their corresponding full-text articles derived from the IntAct and MINT databases. These articles had been curated manually to extract protein interactions for both the interaction pairs as well as the interaction detection methods. We recommended for this subtask not to use articles in the training set describing large-scale experiments (more than 20 to 30 interactions), because they were also excluded from the test set. Not all of the proteins mentioned in a given article are usually studied by all of the mentioned protein interaction detection methods.

As test set for the IPS, IMS and ISS, a total of 358 full-text articles were provided to the participants. The interaction databases MINT and IntAct had previously curated these articles, but held the derived annotations back until the submission phase of test set predictions was over. These articles were provided in the same formats as the training set and resulted from filtering the initial collection provided by the interaction databases following the subselection criteria previously introduced. It was not possible to convert some of the articles to plain text (for instance, PMID 7629138). It was also verified that the overall length and word count of the articles converted to plain text from PDF were consistent with the plain text conversion from the HTML formatted articles.

### Participating methods overview

A common characteristic of the majority of the participating strategies at the IAS was the usage of machine learning techniques (17 out of 19), with SVMs, naïve Bayes, and maximum entropy classifiers being the most frequently used methods. Regarding the natural language processing (NLP) components often integrated into these systems, stemming and POS tagging were the most common ones. Surprisingly, only a few systems exploited Bio-NLP applications such as protein name taggers or adapted existing lexical resources such as biological ontologies for detecting interaction-relevant articles. A number of teams used sentences as their processing unit but most of them based their bag-of-words approaches on whole abstracts as processing unit.

Most of the participating systems did not make use of any additional training data collections to develop their systems, which implies that most of them relied only on the training collections provided by the task organizers. Only a few exceptions can be found, for instance in the case of team 6, who also used a proprietary corpus of biomedical papers annotated with proteins and their interactions.

In addition to MINT and IntAct, other interaction databases are also currently available. The majority of the teams did not exploit annotations derived from these other interaction annotation resources. Some teams had in-house interaction annotation collections, as in the case of team 47, who exploited a collection of their own annotations for system development.

Most of the participating strategies are characterized by the integration of machine learning techniques to address some of the subtasks, with SVMs being the most frequently adapted technique, followed by maximum entropy models.

In order to identify correctly the normalized interactor proteins, it is important to associate text mentions with database records (SwissProt accession numbers). Here the use of protein name tagging and normalization strategies is crucial. The gene mention and gene normalization tasks of the BioCreative challenge addressed these aspects in the case of PubMed abstracts. For the normalization of the interactor proteins from full-text articles, most of the participants used a database look-up and protein name dictionary-based approaches in order to map protein names and symbols contained in the SwissProt database to text mentions. Only a few teams made use of more sophisticated protein mention detection methods like LingPipe [29], Abner [30], or the maximum entropy Markov model based tagger developed by Curran and Clark [31].

In full-text articles, proteins derived from multiple organism sources are often described in the same passage. This is often the case for human proteins and their related mouse homologs. Many protein names contained in biological annotation databases such as SwissProt suffer from interspecies protein name ambiguity, meaning that two proteins from different organism sources share the same name (or symbol). In order to provide correct associations of proteins to SwissProt records, the detection of the corresponding organism source is thus of practical relevance. Not all the strategies used for the PPI task applied organism tagging to improve the interactor protein normalization.

Almost all teams integrated currently available NLP components into their systems for these subtasks. The most frequently used components were POS tagging, stemming, and sentence segmentation algorithms, as well as tokenization and shallow parsing tools. Some systems also used additional elements, such as lemmatization, chunking, and abbreviation extraction (team 6), or predicate analysis (team 49). The following applications were used by one or more teams: Brill's POS tagger, MedPost, Stanford parser, Schwartz and Hearst abbreviation extraction tool, and MxTerminator for sentence segmentation. Only a few teams used external lexical resources such as dictionaries or ontologies. For protein name recognition, team 6 exploited a proprietary protein list derived from RefSeq. A considerable number of strategies were characterized by integrating sentence classifiers to detect interaction-relevant sentences from the full-text articles. Another common feature of the participating strategies was the use of regular expressions or pattern matching strategies (for example, for the tagging of protein or species names as well as for the interaction detection method identification).

## Abbreviations

AUC, area under the receiver operating characteristic curve; BIND, Biomolecular Interaction Network Database; HPRD, Human Protein Reference Database; IAS, interaction article subtask; IMS, interaction method subtask; IPS, interaction pair subtask; ISS, interaction sentences subtask; MINT, Molecular Interactions Database; NLP, natural language processing; POS, part-of-speech; PPI, protein-protein interaction; PSI-MI, Proteomics Standards Initiative Molecular Interaction; SVM, support vector machine.

## Authors' contributions

MK authored the manuscript and was in charge of the task preparation, training set release, test set release and prediction evaluation as well as the result analysis and organization of the evaluation workshop. FL helped in the ISS result analysis and revised the manuscript. CRP revised the manuscript and provided input in the result analysis. AV supervised and coordinated the whole task, participated in the initial task and design and revised the manuscript.
